# The axoneme: the propulsive engine of spermatozoa and cilia and associated ciliopathies leading to infertility

**DOI:** 10.1007/s10815-016-0652-1

**Published:** 2016-01-29

**Authors:** Richard W. Linck, Hector Chemes, David F. Albertini

**Affiliations:** Department of Genetics, Cell Biology and Development, University of Minnesota, Minneapolis, MN 55455 USA; Center for Research in Endocrinology, National Research Council, CEDIE-CONICET, Endocrinology Division, Buenos Aires Children’s Hospital, Gallo 1330, C1425SEFD, Buenos Aires, Argentina; Department of Molecular and Integrative Physiology, University of Kansas Medical Center, Kansas City, KS 66160 USA; The Center for Human Reproduction, New York, NY USA

**Keywords:** Basal body, Fibrous sheath, Intraflagellar transport, Microtubules, Primary cilia, Outer dense fibers

To van Leeuwenhoek who first examined spermatozoa, ca. 1677 [1], and to Gray in 1955 [2] who began to study invertebrate sperm motility, the sperm cell appeared seemingly simple—a head (containing the condensed haploid nucleus) and a flagellum that propels the head to the egg by the propagation of bending waves at nearly 100 Hz in water. By contrast, mammalian spermatozoa are astonishingly complex in their morphology and development from germ cells in the seminiferous epithelia, under the direction of Sertoli cells [3, 4], into their fully formed but functionally inactive state. The inactive spermatozoa then pass through a series of ducts lined by ciliated epithelia followed by maturation in the epididymis. In the female tract, spermatozoa undergo capacitation [5] and self-propulsion through the ciliated oviduct. For reproductive biologists, clinicians, genetic counselors, and general readers, this article will review the advances in our understanding of sperm flagellar and ciliary engines, the axoneme, and some of the defects that cause certain forms of infertility.

## Basic structure of the axoneme from 1888 to the present

**Fig. 1:**
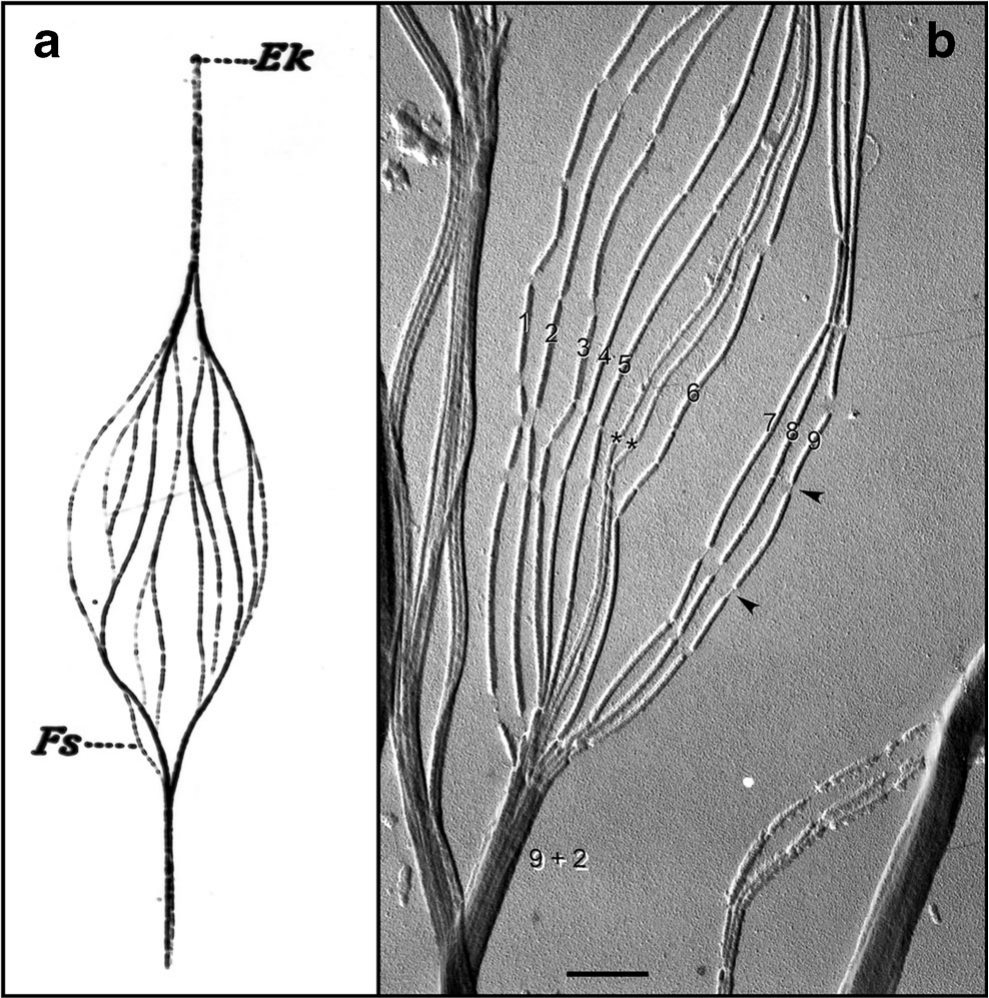
**a** Hand-drawn, light microscopic observations of a splayed rooster sperm flagellum by Ballowitz in 1888 [6], probably the first microscopic examination of sperm flagellar structure, showing the flagellum to be composed of 11 longitudinal elements (*Fs*). *Ek*, Endkörperchen (little end body) probably refers to what is now known as the basal body. **b** Electron micrograph (probably the first) of a splayed rooster sperm flagellum, by Grigg and Hodge in 1949 [7], showing 11 longitudinal elements, nine of which were noted to be wider/denser (numbered) than the other two (*asterisks*). This appearance suggested to the authors that the nine denser elements surrounded the two central less dense elements, also observed and diagrammed by Manton and Clark [8]—i.e., forming what would later be shown by Fawcett and Porter [9] to be the “9 + 2” microtubule axoneme. Grigg suggested to us that the thread-like connections (*arrowheads*) where the microtubule breaksdown (following protease treatment) might correspond to tektin filaments. *Scale bar* (**b**), 0.5 μm. Image courtesy of G.W. Grigg in 1998

**Fig. 2:**
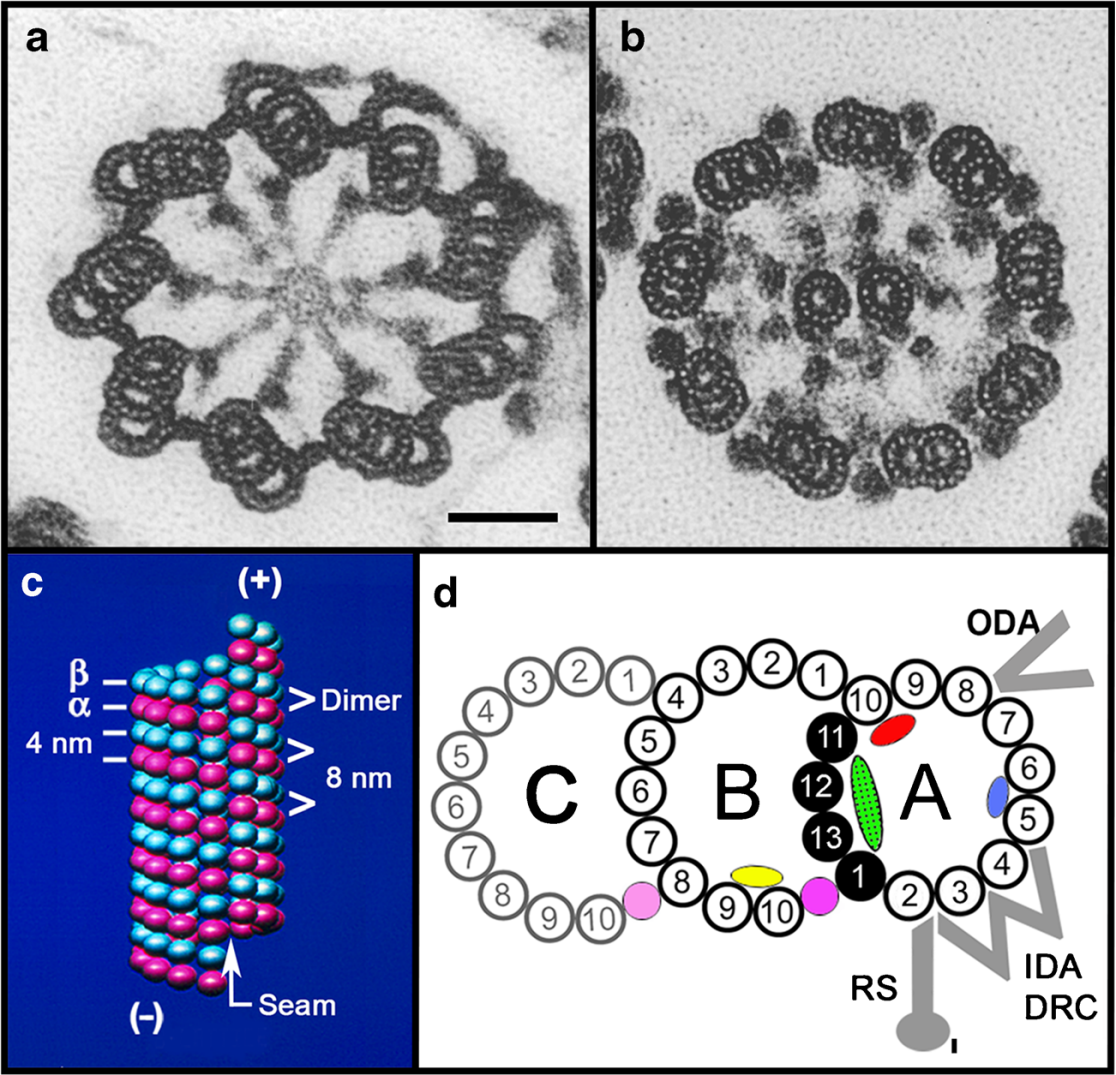
Structure of the basal body (**a**), the flagellar axoneme (**b**), a singlet microtubule (**c**), and a doublet microtubule (**d**). **a**, **b** Electron micrographs of cross sections of tannic acid-stained specimens (an Oxymonad protozoan flagellum) showing the ninefold arrangement of triplet microtubules of a basal body, the 9 + 2 axoneme, and the protofilament substructure of the microtubule walls (compare with Fig. 3). Note: *As viewed from the basal body to the flagellar tip*, the triplet microtubules are tilted in a clockwise pinwheel pattern and the dynein arms point in a clockwise direction toward their adjacent doublet microtubule. *Scale bar* for **a** and **b**, 50 nm; images taken by D. Woodrum Hensley—see [14]. **c** Illustrated are the arrangement and spacings of the α-tubulin and β-tubulin subunits (rendered as spheres) forming the αβ-dimers, their axial repeats (interprotofilament spacing, 5 nm), the lattice or arrangement of subunits around the microtubule wall, the seam or discontinuity in the lattice, and the plus and minus ends of the microtubule. **d** Diagram of the structure of the doublet and triplet microtubule (basal body C-tubule shaded): protofilaments numbered according to convention [15]; the Sarkosyl-insoluble Ribbon of four protofilaments (*black*) [16]; connections of the B-tubule to the A-tubule and the C-tubule to the B-tubule, as determined in **a** and **b**; microtubule inner proteins include MIP1 (*blue*), MIP2 (*red*), MIP3 (*yellow*), inner A-B junctional protein (*pink*), and inner B-C junctional proteins (*faint pink*) [10, 11]; partition-associated material (*green*) [16]; and approximate positions of the outer dynein arms (ODA), inner dynein arms (IDA), dynein regulatory complex (DRC), and radial spokes (RS) [12, 13]

**Fig. 3:**
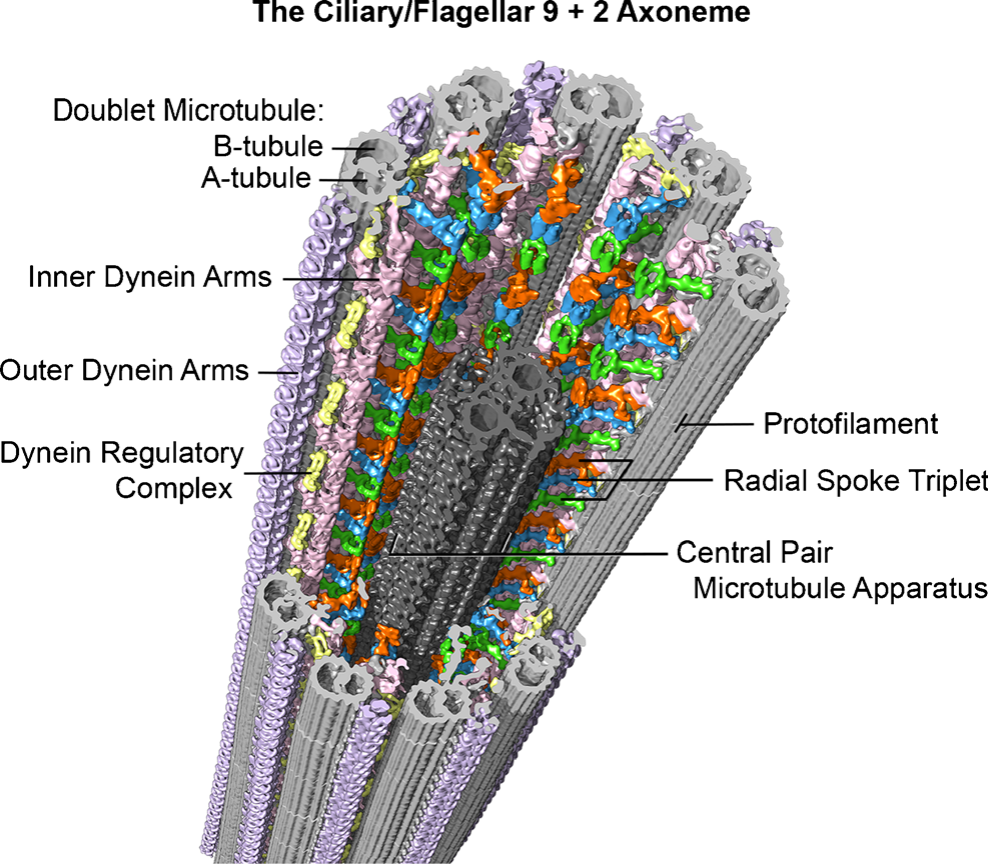
Example of the current, advanced imaging of the 9 + 2 axoneme (from sea urchin, *Strongylocentrotus purpuratus*, sperm flagella), using cryo-electron tomography with a resolution of approximately 3 nm. In this method, isolated flagella or axonemes, applied to special EM grids, are frozen within a few milliseconds in liquid ethane, which prevents damaging ice crystal formation. The specimen is then transferred to a cryo-transfer holder cooled with liquid nitrogen and inserted into the transmission electron microscope. After locating a promising area of a frozen flagellum or axoneme at medium magnification, a tilt series with up to 100 tilted views (from −65° to +65°) is recorded at higher magnification with low electron doses to minimize specimen radiation damage. The tilt series are then computationally aligned and the 3D structure of the specimen is reconstructed. The 96-nm longitudinal repeats of the axoneme (see text) are then extracted and averaged to increase the signal to noise ratio and thus resolution. Finally, the averaged repeat is visualized in 3D using isosurface rendering, as shown here. Some of the major structural features are labeled: Doublet A- and B-tubules (*gray*), radial spokes 1–3 (*green*, *blue*, *orange*), outer dynein arms (*lavender*), inner dynein arms (*pink*), nexin-dynein regulatory complex (*yellow*), and the central pair microtubule apparatus (*charcoal*). Image courtesy of Daniel Stoddard and Dr. Jianfeng Lin from the laboratory of Dr. Daniela Nicastro (Brandeis University and University of Texas Southwestern Medical Center). See references [10–13]

The first investigation of sperm flagellar morphology was begun in 1888, by German cytologist Ballowitz [6], who observed using light microscopy and mordant stains that a rooster sperm flagellum could be splayed into as many as 11, longitudinal fibrils (Fig. 1a) [7–9]. About 60 years later, Grigg and Hodge in 1949 [7] and a year later Manton and Clarke [8] observed these 11 fibers in splayed flagella by electron microscopy (EM) (Fig. 1b); these investigators proposed that two thinner fibers were surrounded by nine thicker outer fibers. In 1952, using advancements in fixation, embedding, and ultramicrotomy, Fawcett and Porter [9] proved by EM thin sections that the core of epithelial cilia within the ciliary membrane consisted of nine doublet microtubules surrounding two central, singlet microtubules (i.e., the “central pair microtubule apparatus”), and hence the term, the “9 + 2” axoneme—Fig. 2b [10–16]. Because of the high degree of evolutionary conservation between cilia and flagella from most species, our understanding of sperm flagella has been aided by studies of both organelles and from species ranging from protists to mammals. Cilia are typically short (5–10 μm) and beat in an oar-like fashion with an effective stroke followed by a recovery stroke [17]. Flagella beat with a snake-like motion and are typically longer (generally 50–150 μm, but ranging from 12 μm to several mm in some species), with flagellar length in the protist *Chlamydomonas* being regulated by several genes encoding kinases [18]. It was recognized first by Manton and Clarke [8] that the 9 + 2 axoneme was possibly ubiquitous among species, and indeed, the nine doublet microtubules are evolutionary conserved structures that evolved in early eukaryotes nearly a billion years ago [19]; however, there is wide variation among species with regard to the detailed structure of sperm flagella and their accessory structures [20]. Axonemal doublet microtubules assemble from the ends of nine centriolar/basal body triplet microtubules [21, 22] (see Fig. 2a), whose ninefold symmetry and clockwise pinwheel pattern (looking from inside the cell to the flagellar tip) is organized by the conserved protein of the SAS6 gene [23], and which is introduced into some eggs to establish the first mitotic spindle. The nine doublet microtubules are then connected around the axoneme by nexin links [24]. Currently, the molecular structure of the axoneme is known to an extraordinary resolution of <4 nm (Fig. 3) through the use of cryo-electron tomography (cryo-ET), as initially pioneered by Nicastro [10, 12]. Sperm flagellar (and ciliary) motility has been effectively analyzed in simple systems (e.g., protist flagella and sea urchin sperm), whose flagella contain several hundred polypeptides by proteomic analysis [25, 26].

## The flagellum of the mammalian spermatozoon

**Fig. 4:**
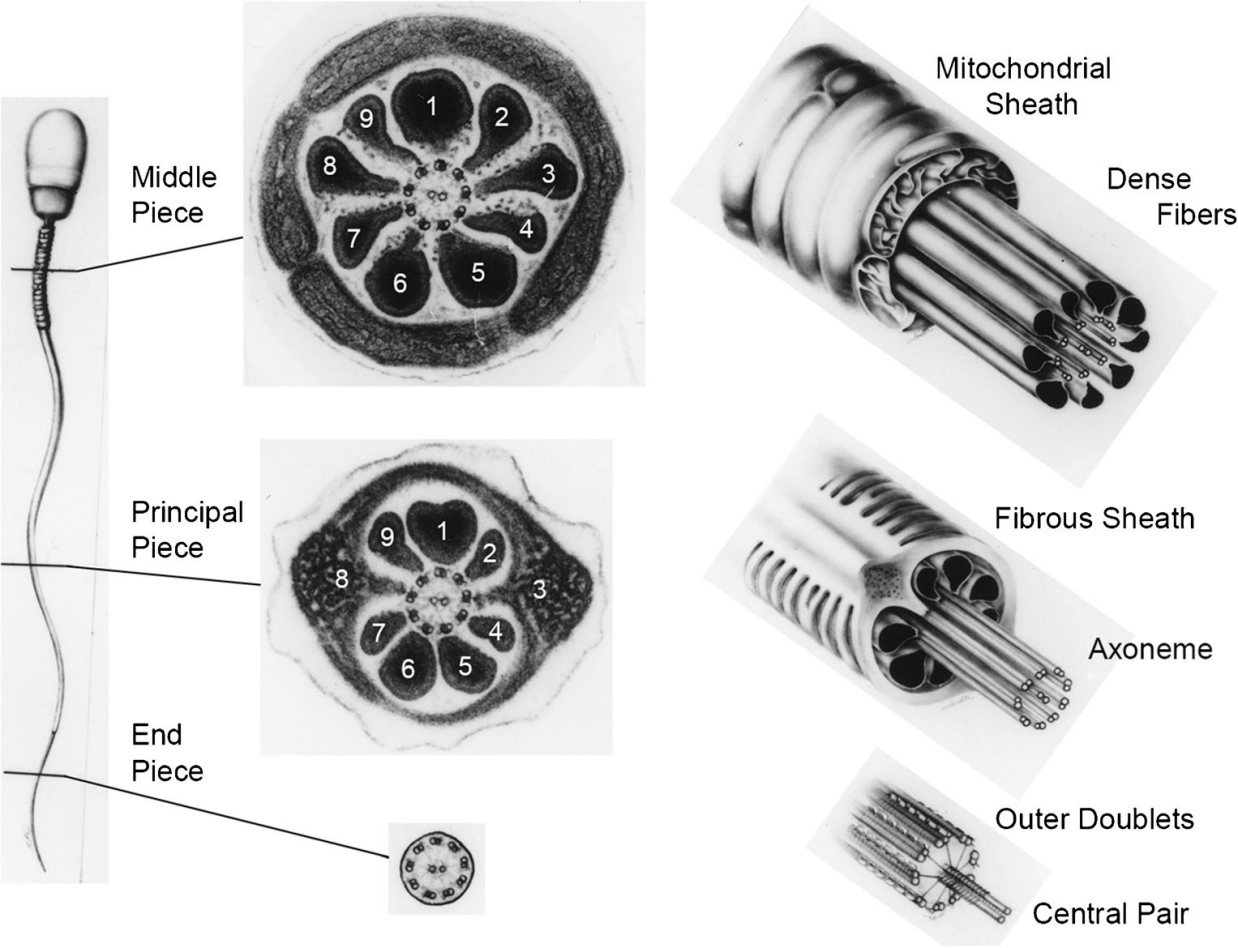
Electron micrographs of cross sections and diagrammatic 3D views of the mammalian (human) spermatozoon, showing the features and transitions occurring along the flagellum. Not illustrated is the fact that the outer dense fibers (ODFs) and the fibrous sheath (FS) gradually taper and terminate where the endpiece extends. Because of the unique morphologies of the ODFs, they and the outer doublet microtubules (to which the ODFs are attached) can be unequivocally numbered. Note also that doublet microtubules #3 and #8 are firmly anchored to their respective longitudinal columns of the FS. Courtesy of Dr. D.W. Fawcett. See references [27, 28]

The flagellum of mammalian spermatozoa is known to be highly complex (Fig. 4), based mostly on the studies of Fawcett [27, 28] (see also [20]), and has a more complex proteome than simple flagella, not even counting the genes and proteins involved in regulating their development [4]. Within the flagellar membrane (whose membrane protein complexity changes along the flagellar length) the axoneme (approximately 50 μm long in humans) lies at the center of (i) *a midpiece*, formed by a sheath of mitochondria wrapped around nine morphologically distinct outer dense fibers (ODFs), each connected to its respective axonemal doublet microtubule, and (ii) *a principal piece* with ribs of the fibrous sheath (FS) surrounding the dense fibers and the two longitudinal columns of the fibrous sheath replacing ODFs #3 and #8. The axoneme protrudes some distance as the *endpiece*. The midpiece itself is anchored to the sperm head by the connecting piece. Invertebrate sperm of octopus and squid also possess ODFs (but not the FS), which appear to provide a necessary function in internally fertilizing animals [29]. Because of the unique shape of each mammalian ODF, they and their respective doublet microtubules can be numbered unequivocally (Fig. 4). The major proteins forming the FS and ODF have been characterized [30–32], and quite interestingly, ODF2 is a homologue of cenexin, a protein associated with the older mother centriole [33, 34]—an evolutionarily earlier protein used by a later cell for a new purpose. The crucial function of these periaxonemal elements and their pathologies will be discussed following a review of the axoneme.

## Molecular structure of the axonemal microtubules

To understand the structural arrangement of the molecular machinery of motility, it is important next to review the structure of the underlying microtubules. In 1973, Tilney et al. [35] demonstrated by thin-section EM that singlet microtubules and sperm flagellar A-tubules of nearly all species are composed of precisely 13 longitudinal protofilaments (Fig. 2). In rapid succession but not chronological order, microtubule protofilaments were shown to be end-to-end polymers of heterodimers of GTP-binding α- and β-tubulin, each with a mass of approximately 50 kDa [36, 37], that could be polymerized into synthetic microtubules in vitro, but with variable numbers of protofilaments ranging from 11 to 17 [38, 39], and off the ends of isolated centrioles [22]. Based on the evolutionarily conserved primary sequence of tubulin [40], the 3D structure was determined for the αβ-tubulin dimer from zinc-tubulin sheets [41]. Tubulin is posttranslationally modified in functionally important ways, e.g., acetylation, glutamylation, glycylation, and tyrosination, which function in the formation of flagellar doublet microtubules [42]—see below. Microtubules are functionally polar structures, as shown by Allen and Borisy [43], with the distal end (subsequently referred to as the “plus” end of the microtubule) assembling tubulin faster in vitro than the proximal (minus) end. The accepted structural polarity of the microtubule is such that the α-tubulin subunit of a dimer is oriented toward the minus end, and the β-subunit toward the plus end, as inferred from Fan et al. [44], where the minus ends, but not the plus ends, can be labeled with a phage display antibody specific to α-tubulin.

Ciliary and flagellar doublet microtubules are significantly more complex than singlet microtubules both in structure and protein composition (Figs. 2 and 3). Early on, they were noted to be the most stable class of microtubules [45], and in nearly all cases, sperm flagellar doublet microtubules are irreversibly assembled into long, elastic elements. Each doublet microtubule is composed of a complete, 13-protofilament A-microtubule (like most cytoplasmic, singlet microtubules) and a partial 10-protofilament B-tubule, which assembles off the ends of the A-B-tubules of the basal body triplet microtubules [21, 35]. The numbering of these protofilaments is formally specified (Fig. 2d), due to their unique positions for the attachments of different motor and regulatory proteins [15]. Using the technique of optical diffraction of electron micrographs, Amos and Klug [46] and Linck and Amos [47] analyzed the arrangement (or lattice) of tubulin subunits in flagellar doublet microtubules, describing the 4- and 8-nm axial repeats of the monomers and dimers, respectively, the 5-nm lateral spacing between protofilaments, the 3-start left-handed helix of monomers, and the lattice of tubulin dimers in the incomplete B-tubule. The lattice of the A-tubule remained uncertain. Recently, Maheshwari et al. [48] showed that the A-tubule has the same lattice as the B-tubule and cytoplasmic microtubules (Fig. 2c); currently, all microtubules in vivo are believed to have the “B-lattice.” Like cytoplasmic singlet microtubules, the A-tubule contains a longitudinal “seam” or helical discontinuity in the lattice (Fig. 2c), with the seam positioned at the outer junction of the A- and B-tubules, i.e., between protofilaments A9 and A10 or between A10 and A11 [48]. The potential functions of the seam in the assembly of microtubules had been discussed earlier [49], but they have not been directly investigated. Doublet microtubules are highly stable and contain a high level of acetylated tubulin (cf. [16]). In addition, the formations of the inner and outer junctions of the A- and B-tubules are affected by posttranslational glutamylation and glycylation of tubulin [42]. In another example, mutations in Arl13b, a G-protein in the Sonic hedgehog signaling pathway, directly or indirectly inhibits the closure of the inner A-B junction in embryonic primary cilia [50, 51]. It is presently unknown to us, whether mutations in these modifications and pathways affect sperm axoneme structure and motility and lead to infertility, in part because the phenotypes are usually lethal.

**Fig. 5:**
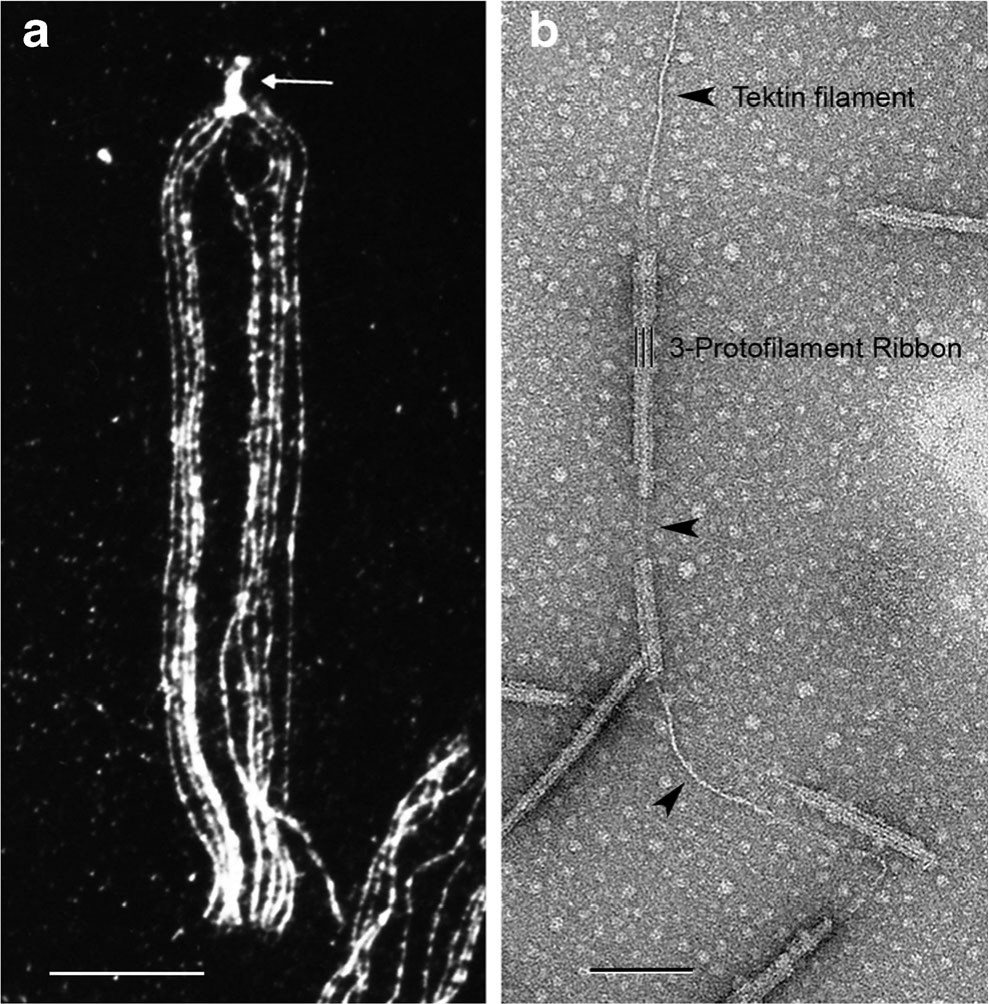
**a** Immunofluorescence light micrograph of a splayed sea urchin sperm flagellum (*L. pictus*) stained with anti-tektin-B antibody, showing the presence of tektin along all nine doublet microtubules (where the punctate staining is due to the masking of tektin epitopes by fixed tubulin) and a greater intensity of tektin staining in the basal body (*arrow*); image taken by W. Steffen [54]. **b** Electron micrograph of a partially fractionated Ribbon of three protofilaments (three black lines) of a sea urchin (*S. purpuratus*) sperm flagellar doublet microtubule, negatively stained, showing the single tektin filament (*arrowheads*) emerging from the end of the Ribbon and/or connecting segments of a disassembled Ribbon; image taken by A. Schefter in the laboratory of R.W. Linck—see [16]. The tektin filament may correspond to the thread-like connections in Fig. 1b. *Scale bars* 10 μm in **a** and 100 nm in **b**

Perhaps underlying their high degree of stability, doublet microtubules from species ranging from protists to mammals contain a single “Ribbon” of three adjoining protofilaments that are resistant to solubilization by 0.5 % sodium dodecyl sarcosinate detergent [52, 53]—Figs. 2d and 5b [54]. The Ribbon is part of the A-tubule wall and lies approximately between the two B-tubule attachment sites [16]. Associated longitudinally with the stable protofilament Ribbon of sea urchin sperm doublet microtubules is a single, hyper-stable, 5-nm filament composed of the fibrous (coiled-coil) proteins, tektins A, B, and C, each approximately 50 kDa in mass and in equimolar amounts; tektins were first cloned and characterized from sea urchin embryonic cilia and sperm flagella, and then from mouse testis meiotic germ cells [16, 55–57]. Tektins extend along the length of each doublet microtubule and into the basal body (Fig. 5a; [54, 58]). Attached to the Ribbon are two calcium-binding proteins involved in juvenile myoclonic epilepsy (cf. [16, 59]).

Despite much information about the biochemistry, structure, and developmental expression of tektins in ciliogenesis and spermatogenesis [60–62], their function remains elusive. Nevertheless, a thorough investigation by Tanaka and colleagues [63] showed that, in mice with a mutation in one of the tektin genes, the males were infertile and the sperm had defective motility and lacked some or all of the dynein arms (see below). There are also several reports of tektin mutations or a reduction in the amount of tektin in mouse [64] and human sperm [65–68] being linked to asthenozoospermia (reduced sperm motility) or oligoasthenozoospermia (asthenozoospermia with a low sperm count), whereas one report found no proteomic difference in tektin levels in normozoospermic and asthenozoospermic humans [69]. Several studies have also reported tektins to be localized to several mammalian sperm structures (refer to Fig. 4) by immunofluorescence microscopy, including (i) the principal piece containing the ODFs and FS and postacrosomal region [70], (ii) the principle piece and the basal body region but absent from the midpiece, and (iii) the endpiece containing only the axoneme, where presumably tektins should be found [71], and from the neck region to the tail piece but stronger in the midpiece (containing the mitochondrial sheath and ODF) [72]. In fact, our own observations (unpublished) showed an intense staining of the FS of rat spermatozoa, using affinity-purified antibodies against sea urchin tektins. While tektins may be components of some of the periaxonemal elements, it seems highly unlikely that tektins would be present in all of the sperm structures. There remains the possibility of strongly binding, nonspecific anti-tektin staining of the accessory structures, and only weak or masked (nondetected) anti-tektin staining of the axoneme, or the presence of other proteins with similar epitopes as tektins (e.g., coiled-coil domains). A proteomic analysis also suggested that tektins may be present in the SDS-insoluble fraction of sperm flagella (e.g., ODFs and FS), but tektin filaments are known to be highly insoluble in metazoan cilia [24].

Axonemal doublet microtubules are the scaffold upon which the protein machinery for motility is attached and with which other proteins interact. Nevertheless, doublet microtubules are not passive elements: they undergo dynamic bending and twisting at acute angles, requiring enormous conformational changes in tubulin, and its associated proteins. There are generally two classes of the motile protein machinery: microtubule motors and regulatory structures and proteins.

## Axonemal dynein motor proteins

The first class of microtubule motors, the dynein ATPases, was discovered and named by Gibbons [73] and shown to form the outer and inner arms observed earlier by Afzelius [74]. Many cases of male infertility involving loss of sperm motility are caused by mutations in the genes for dynein and dynein-associated polypeptides [75–78]. Dynein arms are essentially permanently anchored to each A-tubule and directed to the B-tubules of their next doublet microtubules in a clockwise direction to a viewer looking from the flagellar *base to the tip* [21] (Fig. 2), giving the axoneme enantiomorphic asymmetry or handedness. The importance of this asymmetry to the direction of ciliary motion (to be discussed) and its role in embryonic development may sometimes be misunderstood, as in articles where the handedness is incorrectly diagrammed as being clockwise when viewed from the *tip to the base*, e.g., [76]. For species in which the central pair microtubule apparatus does not rotate (see below), this clockwise handedness allows for the unambiguous numbering of doublet microtubules around the axoneme (see also Fig. 4), with a line perpendicular to the plane of the fixed central pair passing through doublet #1, and between doublets #5 and #6. Since their discovery, dyneins have been studied to the greatest degree in *Chlamydomonas*, which possesses 16 genes for dynein heavy chain polypeptides [79, 80], with the masses of the heavy chains being approximately 500 kDa. In this organism, mutants with motility defects (typically called *pf*-mutants, for paralyzed flagella) can readily be generated and analyzed biochemically and structurally to identify the molecular defects, and therefore, their functions. The outer arms appear to be identical, with each arm (with a mass of ~1.2 mDa) consisting of three dynein heavy chain subunits in protist flagella and two heavy chains in vertebrate sperm, arranged along the A-tubule (for the sea urchin axoneme, see Fig. 3) [12]. Each dynein arm also contains several intermediate and light chain polypeptides. The inner arms are more complex, consisting of a series of 11 dynein heavy chains and associated polypeptides, arranged in a complex but repeating pattern along the A-tubule (Fig. 3 and below).

The mechanism of dynein-based motility was determined in a series of studies beginning with Satir, who showed that in ciliated epithelia, that were rapidly fixed during active beating, the doublet microtubules are preserved in positions where they have slid past each other [81]. Gibbons and colleagues then demonstrated that this sliding was actively driven by dynein ATPase motors, by first demembranating the sperm with mild Triton detergent [82, 83] and digesting the axonemes with trypsin to break the presumably elastic connections between doublet microtubules [84]. Sale and Satir [85] followed by showing that the sliding was unidirectional, with the arms tightly anchored to their A-tubule moving in a minus-end direction along the adjacent B-tubule of the next doublet tubule; hence, the definition of dyneins as being minus-end-directed motors. Brokaw elegantly demonstrated sliding and measured the sliding oscillations in demembranted, reactivated sea urchin sperm by attaching gold particles to the exposed doublet microtubules and observing the oscillations of the particles as the sperm were swimming [86]. Another dynein, involved in a different form of flagellar motility, will be discussed later.

Dyneins are mechanochemical force-transducing enzymes that convert the energy from ATP hydrolysis into a shear force between adjacent doublet microtubules. The dynein cross-bridge cycle has been studied biochemically [87] and by cryo-electron tomography [88], which captures the conformational states of the dynein arm in its cycle. In general, dynein arms anchored along one A-tubule bind ATP, which dissociates them from their prior attachments (cross-bridges) to the B-tubule of the adjacent doublet tubule. After hydrolysis, the dynein arms rebind to the B-tubule. The release of the products (ADP and inorganic phosphate) causes a conformational change in the dynein arm (acting as a lever arm), leading to a shear force, driving the A-tubule in a minus direction along the adjacent B-tubule. In this manner, dynein acts in a manner analogous to muscle myosin interactions with actin filaments. In a mechanism not fully understood but regulated by other axonemal structures (see below), dynein cross-bridges are presumably sequentially activated along the A-tubule from the base/minus to tip/plus ends of the microtubules (like falling dominos).

Both outer and inner dyneins arms are attached along their A-tubule with unique spacings, and they have unique functions. The outer arms form a single row with arms spaced at 24-nm intervals (their axial periodicity). The inner arms consist of several morphologically distinct subunits arranged in a slightly staggered pattern along the A-tubule, with subrepeats of an overall 96-nm axial periodicity (Fig. 3) [12]. These long repeats and different configurations could explain why dynein arms (especially inner arms) sometimes appear absent or reduced in numbers even in normal human sperm. One of these inner arm subunits is the dynein regulatory complex, discovered by Piperno [89] and studied in great detail since then [12, 90]. The inner dynein arms are the primary motors that generate the amplitude and waveform of the propagated bends, whereas the outer arms appear only to provide additional power to increase the speed of wave propagation [83, 91].

## Axonemal bend formation and propagation

**Fig. 6:**
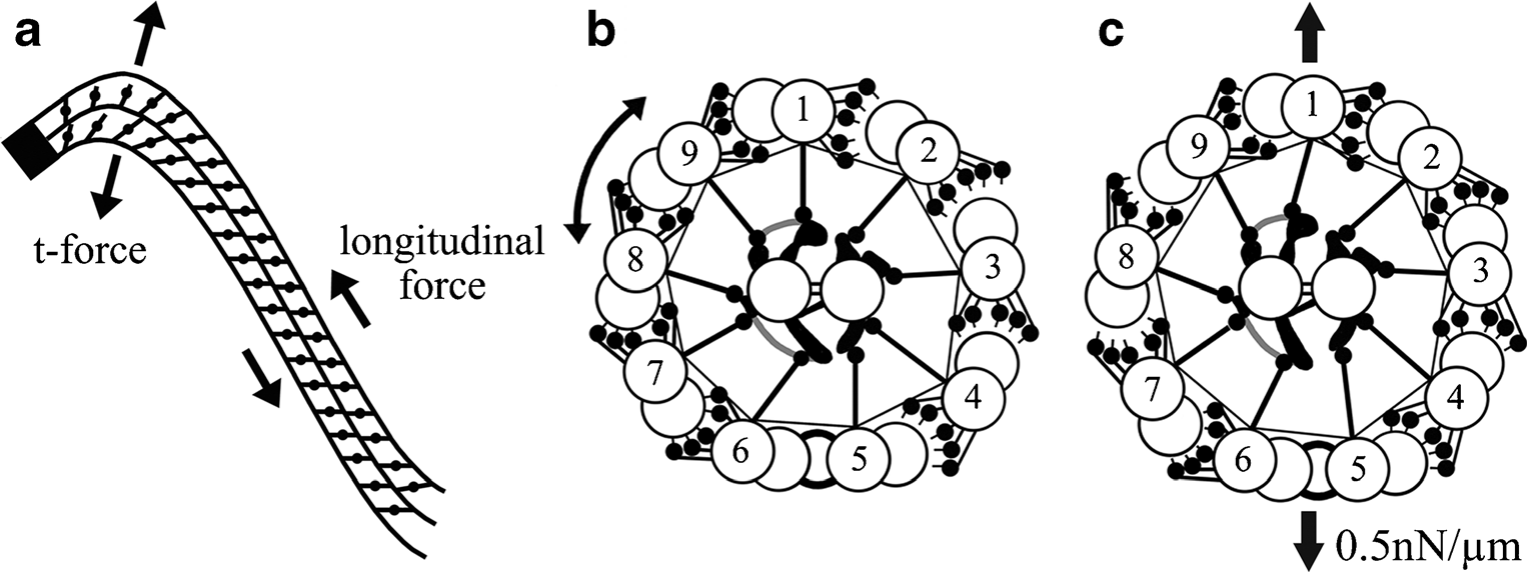
This figure, taken from Lindemann and Mitchell [92], illustrates several features and hypotheses of ciliary and flagellar bend formation and bend propagation. First in **a**, the nine doublet microtubules are anchored to the basal body (*the solid black end*), where they cannot slide initially, but the ability and degree with which they slide immediately increases as the distance from the basal body increases. Secondly, sliding takes place as dynein arms generate longitudinal sliding forces between doublet tubules, moving adjacent doublet tubules toward the base and generating a bend. Thirdly, because of the cylindrical arrangement of the axoneme, the interdoublet sliding will generate a transverse t-force that acts to compress or expand the diameter of axoneme. Finally in **b** and **c**, this t-force will alternately bring doublet tubules on one side of the axoneme closer together and permit dynein-tubule sliding, while on the other side, the doublet tubules are pushed slightly further apart, preventing dynein-tubule interactions; these two events then oscillate back and forth to generate propagated bends. These concepts are brought together in the Geometric Clutch hypothesis [93]. Courtesy of author C.B. Lindemann, and publisher, John Wiley & Sons, Ltd

As the reader can appreciate, the sliding between adjacent doublet microtubules cannot itself produce bend formation, and several other elements are required not only for bend formation but also for the propagation of the bend (Fig. 6) [92, 93]. First, all doublets are anchored to the basal body, where sliding cannot take place initially; sliding only begins a short distance from the basal body, initiating an axonemal bend. Secondly, protein complexes called nexin [24] connect each A-tubule to the next doublet tubule and act as elastic elements to limit the amount of sliding and/or to return the doublets to their resting position. Later, it was with great surprise that nexin was discovered to be part of the dynein regulatory complex of the dynein inner arms, hence the term nexin-DRC [12, 94]. Being one of the subunits of the inner arm repeats, nexin-DRC has a periodicity of 96 nm along the A-tubule (Fig. 3). Thirdly, each A-tubule possesses a firmly anchored, linear row of spokes that radiate inward toward the central pair microtubule apparatus.

## Radial spokes

First distinctly observed by Hopkins [95], radial spokes each consist of a spoke head and stalk and have complex axial spacings [13, 96, 97] (Fig. 3). The absence of radial spokes leads to axonemal paralysis and in sperm, infertility. In *Chlamydomonas* spokes consist of approximately 16 polypeptides [98]. In *Tetrahymena* protozoa, invertebrate cilia, and rat spermatozoa, radial spokes are arranged in a single row with a triplet repeat along the A-tubules, in a polar orientation from the minus to the plus end of the A-tubule,; i.e., a 32-nm spacing between spokes #1 and #2, 24 nm between spokes #2 and #3, and 40 nm between spokes #3 and #1 of the next spoke triplet, all adding up to an overall axial repeat of 96 nm (Fig. 3). In *Chlamydomonas*, spoke #3 appears to be missing, yielding an alternate spacing of 32 nm between the two remaining spokes and 64 nm between spoke pairs; however, it was recently discovered [13] that *Chlamydomonas* does have the basal structure in place of spoke #3 (that was presumably lost in the evolution of that species). Thus, the fundamental axial periodicity of all spoke “triplets” is 96 nm, with radial spoke #2 being in register with the nexin-DRC [12], implying that this registration is essential for the flagellar machinery to work correctly. Radial spokes and their interactions with the central pair microtubules (see below) have been studied in great detail at the molecular level [99–104].

## Evolutionarily conserved axonemal spacings

The evolutionary significance of the longitudinal spacings of the different structures associated with A-tubules is that they are all multiples of the tubulin dimer repeat of 8 nm, i.e., outer dynein arms (24 nm), inner dynein arms (subrepeats of 96 nm), the nexin-DRC (96 nm), radial spokes (32 + 24 + 40 = 96 nm), and in addition, several proteins located along the lumen of the A- and B-tubules [10, 11] repeating at 8 and 16 nm. This means that they must all fit onto the tubulin lattice (Fig. 3); however, *Chlamydomonas* expresses only one α-tubulin isoform and one β-tubulin isoform [105]; thus, tubulin alone probably cannot account for the complex axial spacings nor for the unique pattern of protein structures attached (circumferentially) around the A-tubules. The circumferential asymmetry may be generated by the basal body template but may also be determined by a unique position of asymmetry in the A-tubule itself. Docking proteins have been identified for outer dynein arms, but that only begs the question of how the docking proteins “know” where to attach around the A-tubule. The only known structural-chemical features of the A-tubule wall that might set up a circumferentially asymmetric scaffold are the “seam” positioned at the outer A-B junction [48] and the stable 3-protofilament Ribbon with which the tektin filament is associated [16], but the seam and the position of the Ribbon and tektin filament could in turn be determined by the basal body. As for the complex longitudinal spacings along A-tubules, a ruler protein complex (comprised of FAP59 and FAP172) has been discovered that in *Chlamydomonas* specifies the 32/64-nm axial spacing of the radial spokes and the 96-nm spacing of the nexin-DRC [106]. Curiously, tektins and tektin filaments have many of the axonemal repeats (e.g., 4, 8, 16, and 48 nm) and were speculated to be a ruler [57], but tektins are absent in *Chlamydomonas* mutants that retain the 96-nm radial spoke periodicity [106].

## The central pair microtubule apparatus

The central pair microtubules function to regulate doublet microtubule sliding and potentially taxis and reversal of beat. The absence of the central pair from normally motile cilia and flagella leads to axonemal paralysis and in sperm, infertility; however, they are not essential for motility in all species, because in sperm flagella and motile cilia of some species, the central pair is absent altogether or is replaced by a nonmicrotubule core [20, 107]. Earlier studies of the ultrastructure of the central pair in squid and rat spermatozoa are best summarized elsewhere [29, 97], showing that the two microtubules are structurally different, and that each has two different rows of projections repeating along their respective microtubule with repeats of 16 and 32 nm (again, multiples of the tubulin dimer repeat). The central microtubules and their assemblage of projections act as a fixed unit and are thus referred to as central pair microtubule apparatus (CPMA). More advanced studies with cryo-electron tomography reveal the structure in great detail [104, 108]. As doublet tubule sliding takes place, the radial spoke heads move past and transiently interact with the CPMA, involving phosphorylation and dephosphorylation events that regulate interdoublet sliding, bend formation, and bend propagation [96, 100–104]. Remarkably, the CPMA of protozoa have been shown to actually rotate (i.e., spin) within the nine doublet microtubules, perhaps signaling which and when specific doublet tubules slide [109], and this function could be related to these cilia and flagella being able to reverse the direction of beat. However, in metazoan 9 + 2 cilia (e.g., ctenophore ciliary comb plates which do reverse direction) and spermatozoan 9 + 2 flagella, the CPMA does not rotate [110] and the axonemes initiate their bends and generally beat within a plane that is perpendicular to a line passing through the central pair microtubules, as originally observed by Fawcett and Porter [9]; this feature would seem to produce a more effective propulsive force. Although spermatozoa do not reverse the direction of their beat, they do undergo taxis (see below).

## Regulation of oscillatory bends

The axoneme is a curious and intricate machine. The axoneme appears at first glance to be symmetrical, but as mentioned earlier, it has enantiomorphic asymmetry or handedness, where *viewed from the base to the tip*, the dynein arms point in a clockwise direction toward their adjacent doublet microtubule (Figs. 2 and 3). In some species, certain of the nine doublet microtubules also have specialized structures that the other doublets lack, e.g., a fixed “dynein” bridge between doublet tubules #5 and #6. Furthermore, as mentioned earlier, mammalian spermatozoa possess major accessory structures [20, 27], e.g., ODFs and the FS (Fig. 4), with doublet tubules #3 and #8 anchored to the longitudinal columns of the FS, making doublet tubules #3 and #8 incapable of being moved by their adjacent doublet tubules #2 and #7, respectively (see Fig. 6). These asymmetries and accessory structures manifest themselves in the oscillatory motions of cilia and flagella, which have a principal bend (or effective stroke) and reverse bend (or recovery stroke) [17]. Many cilia and flagella beat in planar waves, but in several species, they beat out of the plane with a helical or semi-helical waveform [17, 111]. Some authors have related the left-handed form of this helical rotation, and the clockwise orientation of the dynein arms, to the direction of fluid flow, e.g., embryonic nodal cilia [112]. However, the correlation between dynein arm orientation, the helical handedness of rotation, and the ultimate effect these might have on ciliary and flagellar function have been called into question in a recent analysis by high-speed holographic microscopy of malaria parasites [113]. Regardless, it is impossible for all doublet microtubules around the axoneme to slide in the same direction simultaneously, and it has been partially demonstrated that the doublets on one side of the axoneme (i.e., doublets 1–4, and their accompanying dense fibers in sperm) slide to produce bending in one direction (while the other doublets are inactive), and that the other doublets (i.e., 6–9) slide to produce bending in the other direction [92]. According to the Geometric Clutch hypothesis [93], these bends appear to generate a transverse force leading to a compression of one side of the axoneme, bringing those doublet microtubules close enough together for their dyneins to actively slide, while the doublets on the other side of the axoneme remain too far apart for dynein-microtubule interactions to occur (Fig. 6); this transverse force oscillates between the two sides of the axoneme to produce propagated bending waves. Computer models take into account as many factors as possible and closely simulate the physical dynamics of flagellar wave propagation [93, 114].

## Sperm taxis

Having a propulsive engine is only useful if it can be guided. Sperm and most ciliated and flagellated protists and metazoans have mechanisms to alter the motion of their axonemes in order to move in a favorable direction, i.e., taxis. These mechanisms include chemotaxis, rheotaxis, thermotaxis, and phototaxis, with the first three occurring in mammalian sperm in order to move toward the ovum, but all four share similar signaling pathways. These topics have been discussed elsewhere and will only be mentioned here briefly. The most well understood are phototaxis in *Chlamydomonas* [115] and chemotaxis from studies of marine invertebrate (e.g., sea urchin) sperm [116], where chemotaxis operates at distances of less than a millimeter and is recapitulated in mammalian sperm [116–119]. When a chemical attractant isolated from eggs binds to receptors on the sperm flagellar membrane, it causes an influx of calcium into the sperm cytoplasm through membrane calcium channels (polycystins?—see below), activated in mammals by progesterone and prostaglandins; mutations in the channel protein result in mammalian infertility [119]. The rate of increase in intracellular calcium (from pCa 8 to pCa4) is followed by a G-protein signaling cascade in conjunction with a brief depolarization of the sperm membrane and a rise in cAMP levels. These signals are transmitted to, and stimulate the axoneme, and evidence implicates phosphorylation of radial spoke/CPMA interactions that relay to the phosphorylation of inner dyneins and nexin-DRC, which according to Lindemann and colleagues [117] may determine which doublet microtubules on which side of the axoneme will slide in a given sequence (Fig. 6); dephosphorylation presumably returns the axoneme to it prestimulated state. Less well understood is how these events alter the direction of sperm swimming. The excellent article by Seifert, Kaupp, Strünker and colleagues [119] suggests that sperm chemotaxis is analogous to bacterial chemotaxis, where in favorable conditions (steady or increasing concentrations of attractant) sperm swim in straight lines, and in less favorable conditions (lower attractant concentrations), they briefly randomize their swimming direction, and repeat this process until a favorable conditions allows them to again swim linearly, e.g., toward the egg. Nearly similar mechanisms are involved in thermotaxis (in the range of 31 to 37 °C) and rheotaxis (movement against a fluid flow), both of which have been identified and tested in mammals, principally mice and rabbits, and in vitro for human sperm [118].

## Intraflagellar transport

In addition to the mechanism of propulsive force for sperm movement (axonemal bends and wave propagation), flagella and cilia have a second form of motility termed intraflagellar transport (IFT), as first reported by the laboratories of Witman, Pazour, Rosenbaum, and Yoder [120–123], and studied more recently in its astonishingly widespread effects on vertebrate development (see below). IFT is independent of propulsive axonemal motility (i.e., it occurs in nonmotile cilia and flagella) but does require the axoneme to be present. IFT involves both a unique form of dynein and a second class of microtubule motors, the kinesins. Kinesin motors (which are plus-end motors) move their specialized protein cargo (“IFP particles” containing ciliary precursors and signaling molecules) from the cell body to the flagellar or ciliary distal tip along the doublet microtubules in the region between them and the membrane, while IFT-dyneins (minus-end motor) move other cargo from the distal tip back to the cell body. IFT, however, has not so far explained how the massive amount of tubulin required for axoneme assembly is transported to the growing distal tip or for tubulin turnover in terminally differentiated axonemes [124, 125]. IFT has been thoroughly investigated in *Chlamydomonas* flagella and vertebrate (mouse) cilia, and is essential for ciliary and flagellar assembly and for signaling pathways (e.g., Hedgehog, PDGF, and Wnt pathways). To our knowledge, IFT has not been studied in mature mammalian spermatozoa; however, it is entirely expected that IFT would be essential for the formation of the mammalian sperm tail during spermatogenesis, and IFT could perhaps function in signaling events during capacitation or later during guided propulsion toward the egg. We are not aware of such studies in mammalian sperm, but in *Drosophila* with a disrupted polycystin-2 gene (see below), the sperm are motile but effectively sterile, because they fail to enter the female sperm storage organ [126]. Finally, it is important to understand that entry of molecules from the cytoplasm into the ciliary/flagellar environment, and their exit from it, are regulated by a cytoplasmic compartment termed the “ciliary gate” or “ciliary pore,” located between the basal body and the plasma membrane [127].

## Ciliopathies: pathologies of cilia and flagella

**Fig. 7:**
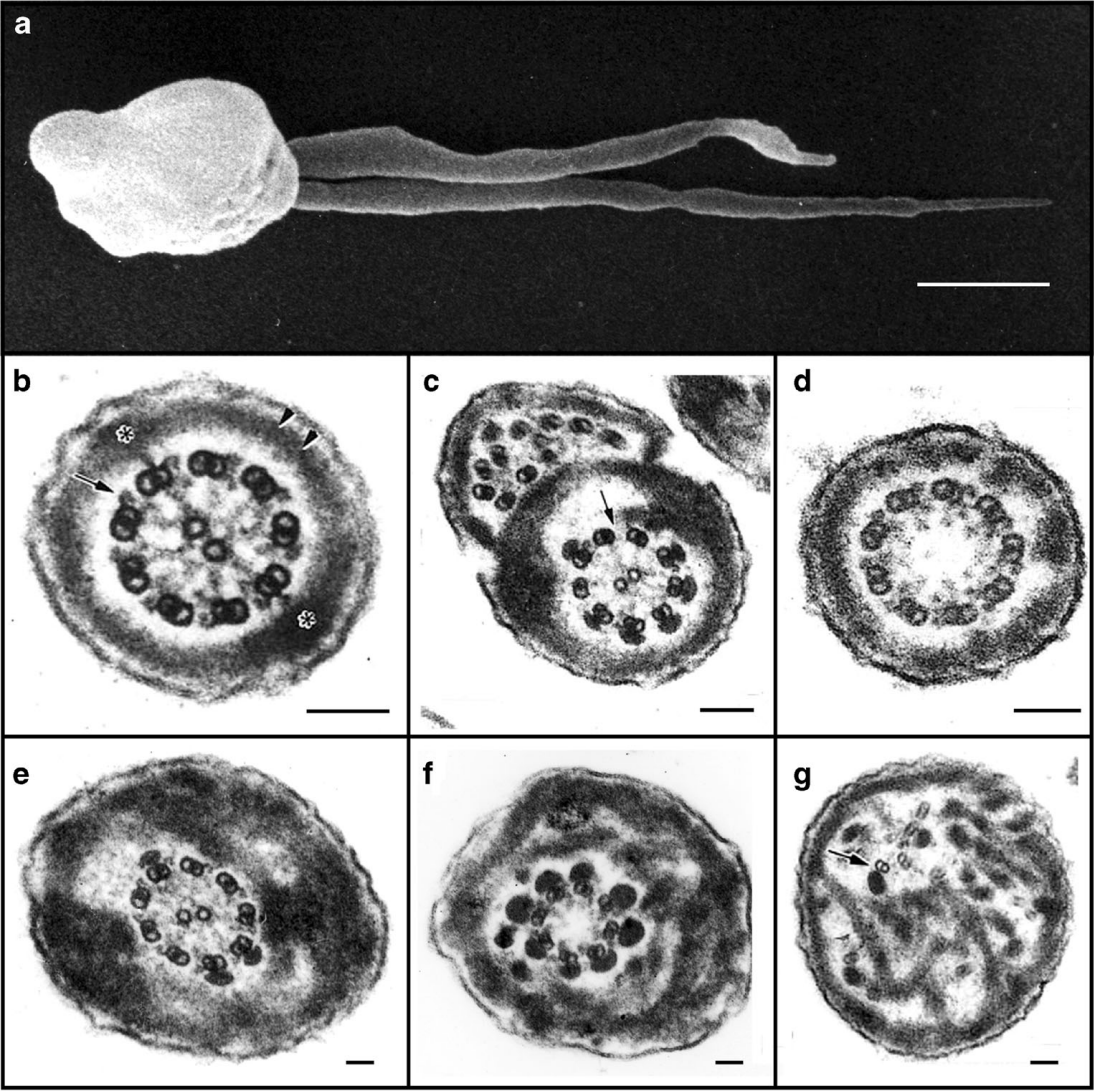
Electron microscopic analysis of human sperm pathologies. **a** Scanning electron micrograph of a dysplasia of the fibrous sheath (DFS) in human spermatozoa. Note the two thick, irregular and very short tails (length ˂10 μm, normal 50–60 μm). **b** Transverse section of a normal flagellum at the distal principal piece (compare with Fig. 4). At this point, the axoneme is composed of nine doublet microtubules around a central pair microtubule apparatus (CPMA), nine radial spokes projecting toward the CPMA, and two dynein arms (outer and inner) anchored to each A-tubule and oriented in a clockwise direction toward the B-tubule of the neighboring doublet microtubule (*arrow*), as viewed from the base to the tip of the flagellum. The FS is composed of two lateral columns inserting into doublet microtubules #3 and #8 (*asterisks*), and two semi-circumferential ribs (*double arrow head*). By this point along the flagellum, the ODFs have terminated. **c**, **d** Spermatozoa from two patients with primary ciliary dyskinesia. In **c**, the fused complete and incomplete axonemes are due to the failure of neighboring spermatids to separate from their cytoplasmic bridges, and here, there is partial lack of dynein arms (*arrow*) and FS distortions. In **d**, the CPMA is missing (i.e., a 9 + 0 axoneme) but radial spokes are still present. **e**–**g** Three transverse sections of DFS spermatozoa with marked FS abnormalities: the FS forms thick disordered periaxonemal rings and the lateral columns are misplaced in **e** and **f**; the axoneme in **e** is preserved, but in **f**, there is lack of one doublet microtubule and the CPMA is missing (8 + 0 axoneme). In **g**, note the complete disorientation of the axoneme, where one doublet microtubule appears to lack dynein arms (*arrow*). Diameters of pathological flagella may range from 1 to 1.2 μm (normal flagellar diameter ≅ 0.4 μm). *Scale bars* 1 μm in **a** and 0.1 μm in **b**–**g**

With several hundred genes and proteins involved in developing spermatocytes and spermatids and in Sertoli cells for the assembly and motility of the spermatozoon axoneme [3, 4], it should be no surprise that genetic mutations, endocrine disruptors, or environmental toxins, will affect the synthesis or activity of these proteins and negatively impact spermatozoon development, motility, and thus fertility. The first genetic pathology affecting human spermatozoa was discovered in 1975, by Afzelius et al. [128] and Pedersen and Rebbe [129], who observed patients with Kartagener’s syndrome, i.e., male infertility coupled with sinusitis, respiratory disorders, and *situs inversus* (including the development of the heart on the right side). They traced this pathology to the absence of axonemal dynein arms leading to the immotility of spermatozoa (see Fig. 7) [130–133] and later to the immotility of respiratory cilia (see below). Afzelius postulated that the potential lack of ciliary motility in the embryo might ultimately affect the rotation of the developing heart [134]. This hypothesis was tested by Hirokawa and colleagues [135], who knocked out the gene for kinesin KIF3B in mice, that is required for IFT and ciliogenesis. The consequences of this knockout were that (a) the normally motile 9 + 0 primary cilia of the embryonic Henson’s node were absent, and (b) 50 % of the embryos developed *situs inversus* (along with many other developmental disorders). Numerous investigators have since shown that in the absence of ciliary formation or motility, signaling does not take place to activate genes that will rotate the developing cardiac sac in the correct (leftward) direction (see below).

The original term suggested for these ciliary and flagellar pathologies was “immotile cilia syndrome” [136], but the term primary ciliary dyskinesia (PCD) was later adopted to cover a wider range of “ciliopathies” of motile cilia [137, 138], including cilia that are not completely immotile or that are abnormally motile. As the name implies, the term PCD is a subcategory of broader ciliopathies, because it does not refer to cases of the complete absence of cilia, or to defects in *normally nonmotile* cilia, e.g., auditory hair cell kinocilia (in deafness), olfactory cilia (in anosmia), retinal photoreceptor connecting cilia (in retinal degeneration), and kidney cilia (in polycystic kidney disease)—see below. What may confuse some readers is that “primary” in PCD does not refer to “primary cilia” (which are “short, single, nonmotile” [139]), but to the fact that PCD is a primary genetic defect, rather than a secondary, acquired condition (note: “nonmotile” refers to the fact that primary cilia do not propagate dynein-depending bending waves; it does not apply to IFP or to deflections of cilia produced by cytoplasmic actin-myosin attached to the basal bodies). To avoid confusion here, we will use the more inclusive term, ciliopathies.

Following the discovery of the cause-effect of missing dynein arms and dynein-associated mutations in nonmotile sperm, additional defects were found not only in human sperm flagella but in human respiratory (and other) epithelial cilia, illustrating common mechanistic denominators between cilia and flagella, but also differences between them. Additional axonemal defects (Fig. 7) include the lack of the central pair microtubule apparatus (CPMA), the lack of radial spokes, missing or transposed doublet microtubules, ciliary aplasia, and the abnormal migration of basal bodies to the apical cell surface [140–144]. The absence of the missing CPMA is noteworthy. While sperm require the CPMA for motility, embryonic nodal cilia have no central pair (i.e., a 9 + 0) but are motile, having a rotary motion that is responsible for left-side rotation of the developing cardiac sac. When nodal cilia are immotile or not present, heart orientation occurs randomly, with about 50 % of patients having their hearts positioned on the right [134]. Thus, it would seem obvious that normally motile 9 + 0 axonemes possess a different mechanism of initiating and regulating axonemal motility than do normally motile 9 + 2 axonemes (e.g., sperm flagella), perhaps due to differences in their nexin-dynein regulatory complexes (DRC)—recalling that suppressor mutants of the nexin-DRC restore motility to nonmotile mutants lacking radial spokes [12, 89, 90]. Since the nexin-DRC complex holds the nine doublet microtubules together [12], it would be worth knowing if nexin-DRC also exists in normally nonmotile cilia (e.g., 9 + 0, nonmotile cilia of the kidney and retina).

Still, some defects present in sperm flagella are not apparent in cilia, and vice versa, reflecting differences between cilia and flagella (e.g., sperm flagella possess periaxonemal structures, while cilia do not), and/or differential regulation of axonemes in somatic and germ cells. Of the various sperm flagellar periaxonemal elements, the ODFs and FS (Fig. 4) do not appear to contribute actively to motility but are thought to dampen the amplitude of flagellar waves for movement through more viscous environments of the oviduct, by allowing longer stretches of dynein arms to generate and propagate bending waves [145]. In addition, the relative orientations of the CPMA, the FS, and the especially thick ODFs #1, #5, and #6, limit the spermatozoan to beat largely in a plane perpendicular to a line through the central pair microtubules and the columns of the FS (Fig. 4); this plane of beat may be more efficient for the propulsion the spermatozoan through the oviduct. In addition, splice variants of ODF2 gene/protein homologous with cenexin are associated with centrioles and essential for centriologeneis and ciliogenesis [33, 34, 146]. While not necessarily active, motile elements, ODF and FS are essential for sperm motility, as demonstrated by ultrastructural defects that occur leading to male infertility [147–150] (Fig. 7). Sperm tails may appeared thick, short, or irregular, or duplicated due to the failure of neighboring spermatids to separate their cytoplasmic bridges. Besides the lack of dynein arms and/or the CPMA (Fig. 7c, d), other anomalies include missing, misplaced, or aberrant doublet microtubules and their associated ODF, e.g., 8 + 0 axoneme (Fig. 7f), and widespread disarray of the axoneme and FS (Fig. 7e–g). The latter phenotype was characterized as dysplasia of the fibrous sheath or DFS [130–133], owing to the remarkable participation of fibrous sheath abnormalities and its origin from a dysplastic development of the sperm tail cytoskeleton. DFS has family incidence, associates to classical forms of PCD, and does not respond to any therapies. A genetic origin was suggested for PCD-DFS (and its combinations) by reports of mutations-deletions in genes encoding axonemal and periaxonemal proteins [151, 152]. Sperm flagellar disorders are unique among ciliopathies, where the periaxonemal structures only appear in the adult, and axonemal defects in the embryo are often lethal or compromise the adult to the point that reproductive function is never reached. Certainly, some of the defects in the periaxonemal structures would be due to mutations in the genes for and regulation of the FS and ODFs, and other defects would be due to defects in the axoneme which acts as a scaffold for the FS and ODF assembly.

Given the structural and functional similarities between sperm flagella and epithelial cilia, and given that the male reproductive tract also contains ciliated epithelia in the rete testis and efferent ducts (as well as in their embryonic progenitor cells), one should ask how defects in these cilia might impact male infertility. There are at least two lines of investigations that address this point. The first of these relates male infertility to polycystic kidney disease (PKD). In the normal kidney specific epithelial cells of uriniferous tubules possess a single, primary, nonmotile, 9 + 0 cilium that projects into the lumens of the uriniferous tubules and collecting ducts. These cilia sense fluid flow and respond via calcium influx through channels containing the transmembrane proteins, polycystin-1, and polycystin-2 [153]; polycystin gene PKD1 also interacts with the Tgf-β/Bmp signal transduction pathway. PKD has been shown to be directly linked to mutations in polycystin genes [154]. In PKD, cilia-based cell signaling is compromised, growth control is lost, and the normal tubular epithelia forms spherical cysts. There is a high correlation between PKD and male sterility. Some human PKD patients have 9 + 0, nonmotile sperm [155], some patients have cysts in the seminal vesicle [156], and in mice with disrupted PKD2 genes malformations occur in the testis, the mesonephric ducts (embryonic origin of the efferent ducts) and the epididymis, with resulting male sterility [157, 158]. The second line of investigation relates male infertility to retinal degeneration, which involves the retinitis pigmentosa GTPase regulator gene (RPGR) and results from a failure of intraflagellar (ciliary) transport (IFT) of newly synthesized material from the cell body along the photoreceptor connecting cilium (a primary, 9 + 0, nonmotile axoneme) to the outer rod segment (see [121]). In transgenic mice overexpressing RPGR, which were sterile, there was either a complete absence of flagella, or there were defects in the assembly and organization of the axoneme, FS and ODFs, indicating a role of RPGR and IFT in sperm tail formation [159]. Oddly, ciliopathies that would be expected to affect oviduct cilia, only slightly impair the female reproductive system [160].

In conclusion, ciliopathies are widespread human diseases and disorders, in many cases affecting male fertility. Most if not all human embryonic cells possess cilia (motile and nonmotile) and most adult cells retain them and the testis makes (sperm) flagella. Currently, the manifestations of ciliopathies are known to include anosmia, bone, cartilage and tooth development, brain development and disease (e.g., hydrocephaly, juvenile myoclonic epilepsy) and many other neurological abnormalities, deafness, hyperphagia, male infertility, obesity, hepatic, pancreatic and splenic diseases, polycystic kidney disease, polydactyly, respiratory diseases, retinal degeneration, and *situs inversus*. Clearly, this information indicates a highly complex set of interacting genetic pathways controlling the assembly of cilia and flagella, their motile functions, and their signaling functions. Mutations occurring downstream may have only a single effect on development (e.g., the sperm flagellar defects and infertility), while other mutations upstream will have global effects (e.g., as in Kartagener syndrome and Bardet-Biedl syndrome), which will inevitably impact sperm development and fertility. The genetic pathways and cell signaling pathways associated with cilia are beyond the scope of this article and readers are referred to the cited literature and earlier reviews [26, 75–77, 127, 161–165].

## Abbreviations

3D: Three-dimensional
CPMA: Central pair microtubule apparatus
FS: Fibrous sheath
IFT: Intraflagellar transport
ODF(s): Outer dense fiber(s)
ODFs #1, #3, #5, #6, and #8: Refer to specific ODFs
Odf2: One of the major proteins of ODFs
PKD: Polycystic kidney disease
PCD: Primary ciliary dyskinesia
